# Dengue Virus Infection of *Aedes aegypti* Requires a Putative Cysteine Rich Venom Protein

**DOI:** 10.1371/journal.ppat.1005202

**Published:** 2015-10-22

**Authors:** Berlin Londono-Renteria, Andrea Troupin, Michael J Conway, Diana Vesely, Michael Ledizet, Christopher M. Roundy, Erin Cloherty, Samuel Jameson, Dana Vanlandingham, Stephen Higgs, Erol Fikrig, Tonya M. Colpitts

**Affiliations:** 1 Department of Pathology, Microbiology & Immunology, University of South Carolina School of Medicine, Columbia, South Carolina, United States of America; 2 Foundational Sciences, Central Michigan University College of Medicine, Mount Pleasant, Michigan, United States of America; 3 L2 Diagnostics, New Haven, Connecticut, United States of America; 4 Department of Tropical Medicine, Tulane University School of Public Health and Tropical Medicine, New Orleans, Louisiana, United States of America; 5 Biosecurity Research Institute, Kansas State University, Manhattan, Kansas, United States of America; 6 Diagnostic Medicine and Pathobiology, Kansas State University, Manhattan, Kansas, United States of America; 7 Section of Infectious Diseases, Department of Internal Medicine, Yale University School of Medicine, New Haven, Connecticut, United States of America; 8 Howard Hughes Medical Institute, Chevy Chase, Maryland, United States of America; University of Massachusetts Medical School, UNITED STATES

## Abstract

Dengue virus (DENV) is a mosquito-borne flavivirus that causes serious human disease and mortality worldwide. There is no specific antiviral therapy or vaccine for DENV infection. Alterations in gene expression during DENV infection of the mosquito and the impact of these changes on virus infection are important events to investigate in hopes of creating new treatments and vaccines. We previously identified 203 genes that were ≥5-fold differentially upregulated during flavivirus infection of the mosquito. Here, we examined the impact of silencing 100 of the most highly upregulated gene targets on DENV infection in its mosquito vector. We identified 20 genes that reduced DENV infection by at least 60% when silenced. We focused on one gene, a putative cysteine rich venom protein (SeqID AAEL000379; CRVP379), whose silencing significantly reduced DENV infection in *Aedes aegypti* cells. Here, we examine the requirement for CRVP379 during DENV infection of the mosquito and investigate the mechanisms surrounding this phenomenon. We also show that blocking CRVP379 protein with either RNAi or specific antisera inhibits DENV infection in *Aedes aegypti*. This work identifies a novel mosquito gene target for controlling DENV infection in mosquitoes that may also be used to develop broad preventative and therapeutic measures for multiple flaviviruses.

## Author Summary

Dengue virus (DENV) is responsible for serious human disease worldwide and the World Health Organization estimates that over 2 billion people are at risk for disease. There are no vaccines or specific antiviral medications currently available for DENV infection. DENV is transmitted to humans by infected mosquitoes during feeding and probing. By examining the effects of virus infection on gene expression, and interactions between virus and vector, we may be able to find new targets for prevention and treatment. Here we look at a mosquito protein, CRVP379, whose gene expression was highly increased during DENV infection in mosquitoes. We show a requirement for CRVP379 during DENV infection in the mosquito and a correlation between the levels of CRVP379 and levels of infection. Our results indicate that the protein may be acting with a putative DENV receptor in the mosquito, prohibitin protein. These data also suggest that blocking CRVP379 function may be used to block DENV infection in the mosquito.

## Introduction

Dengue virus (DENV) is the most important arbovirus in tropical areas leading to substantial pediatric morbidity and mortality worldwide [1–6]. DENV is transmitted to humans via the bite of an infected mosquito of the *Aedes spp*. Infection with DENV in humans can result in dengue fever (DF), dengue shock symptom (DSS) and dengue hemorrhagic fever (DHF), the latter two that can lead to severe disease and death. There are no specific antivirals or approved vaccines for use in DENV treatment or prevention [4,7–9]. Current dengue control methods rely mostly on activities to reduce vector population [10,11]. The increase in number of cases despite vector control indicates that these strategies are not as effective as expected, and that new tools need be developed to alleviate disease burden in endemic areas [12–14].

During the last five decades, much effort has been invested in the development of vaccines against DENV [15–23]. One of the obstacles in dengue vaccine development is the potential risk of severe disease mediated by the presence of sub-neutralizing antibodies against virus particles. These antibodies can predispose an individual to severe disease through a phenomenon called antibody-dependent enhancement (ADE), where the virus can infect cells via FcR in mononuclear cells [8,9,24–28]. Traditional vaccine approaches have included live attenuated viruses, recombinant subunits, virus-like particles and plasmid or viral vectors. There are live attenuated and chimeric DENV vaccines that have gone into clinical trials but none have proven to provide complete and lasting protection against all four DENV serotypes [21,29].

An attractive complement to traditional vaccines is to induce an immune response in the vertebrate host (infected or non-infected) that will block virus infection of the mosquito vectors. This would successfully interrupt transmission by inducing antibody responses against non-viral antigens [30]. These type of vaccines are called transmission-blocking vaccines (TBV), since they aim to interfere with pathogen development within the vector, thereby blocking transmission to human hosts [31]. The majority of TBVs designed to inhibit malaria infection are based on the mammalian immune response to pathogen proteins [31]. Another category of TBVs in development are based on arthropod molecules able to reduce pathogen infection in vector tissues [32]. For arboviruses, vector molecules able to interact directly with the pathogen (i.e. ligands/receptors) are highly suitable candidates for blocking transmission [33,34].

The main global transmission vector for DENV is *Ae*. *aegypti*. Extensive research has shown that DENV infection of *Ae*. *aegypti* induces many varied changes in gene expression [35–44]. Our hypothesis is that genes upregulated during DENV infection are required for virus survival or are related with defense against infection [37]. Consequently, a better understanding of the role of mosquito proteins regulated by DENV infection will reveal important insights into DENV biology and transmission as well as be helpful to the design of an effective TBV against DENV. For example, antibodies directed against mosquito molecules involved in steps of the pathogen life cycle are promising candidates for TBV. In addition, a recent study demonstrated that antibodies against a mosquito C-type lectin, mosGCTL1, effectively interrupts the infection of *Ae*. *aegypti* mosquitoes with DENV [34]. Other proteins which genes are unregulated upon infection also show promising capacity of interrupting infection since they are considered important for the microorganism survival. One of these proteins is the tick histamine release factor (tHRF) from *Ixodes scapularis* upregulated during *Borrelia burgdorferi* infection. Previous work showed that expression of tHRF is associated with the tick blood feeding and that the silencing its gene by RNA interference or antibodies not only effectively impairs tick feeding but subsecuently decreases *B*. *burgdorferi* burden [45].

Using comprehensive microarray analysis to identify key alterations in the *Ae*. *aegypti* transcriptome during flavivirus infection, we previously identified 203 mosquito genes that were up- and 202 genes that were down-regulated during infection [35]. Comparative analysis revealed that at least 15 of these genes had differential expression during infection with DENV, Yellow fever (YFV) and West Nile virus (WNV) [35]. One of these conserved, up-regulated genes was a putative cysteine-rich venom protein (AAEL000379), which we named CRVP379. Cysteine-rich venom proteins (CRVPs) are expressed in multiple mosquito tissues including the salivary glands [37,46,47]. Examples of mosquito CRVPs include an *An*. *stephensi* peptide annotated as salivary-secreted serine protease inhibitor [48] and a putative cysteine-rich protease inhibitor found in the sialotranscriptome of adult female *Culex quinquefasciatus* [49]. The specific role of these proteins in mosquitoes remains unknown [46,47].

Here, we describe a requirement for CRVP379 during DENV infection in mosquito cells and live mosquitoes, including a direct correlation between the amount of CRVP379 expressed and the level of DENV infection. We demonstrate the importance of an interaction between CRVP379 and prohibitin, a putative DENV receptor protein in mosquitoes. We also assess the tissue-specific expression of CRVP379 during DENV infection. Finally, we use both RNAi and specific antibody to demonstrate that blocking CRVP379 results in inhibition of DENV infection in *Ae*. *aegypti*. These results further our understanding of DENV pathogenesis in the mosquito vector and highlight a potential target protein for the creation of a DENV TBV to break the host-vector transmission cycle.

## Results

### Silencing virally up-regulated genes alters DENV infection in mosquito cells

**Fig 1:**
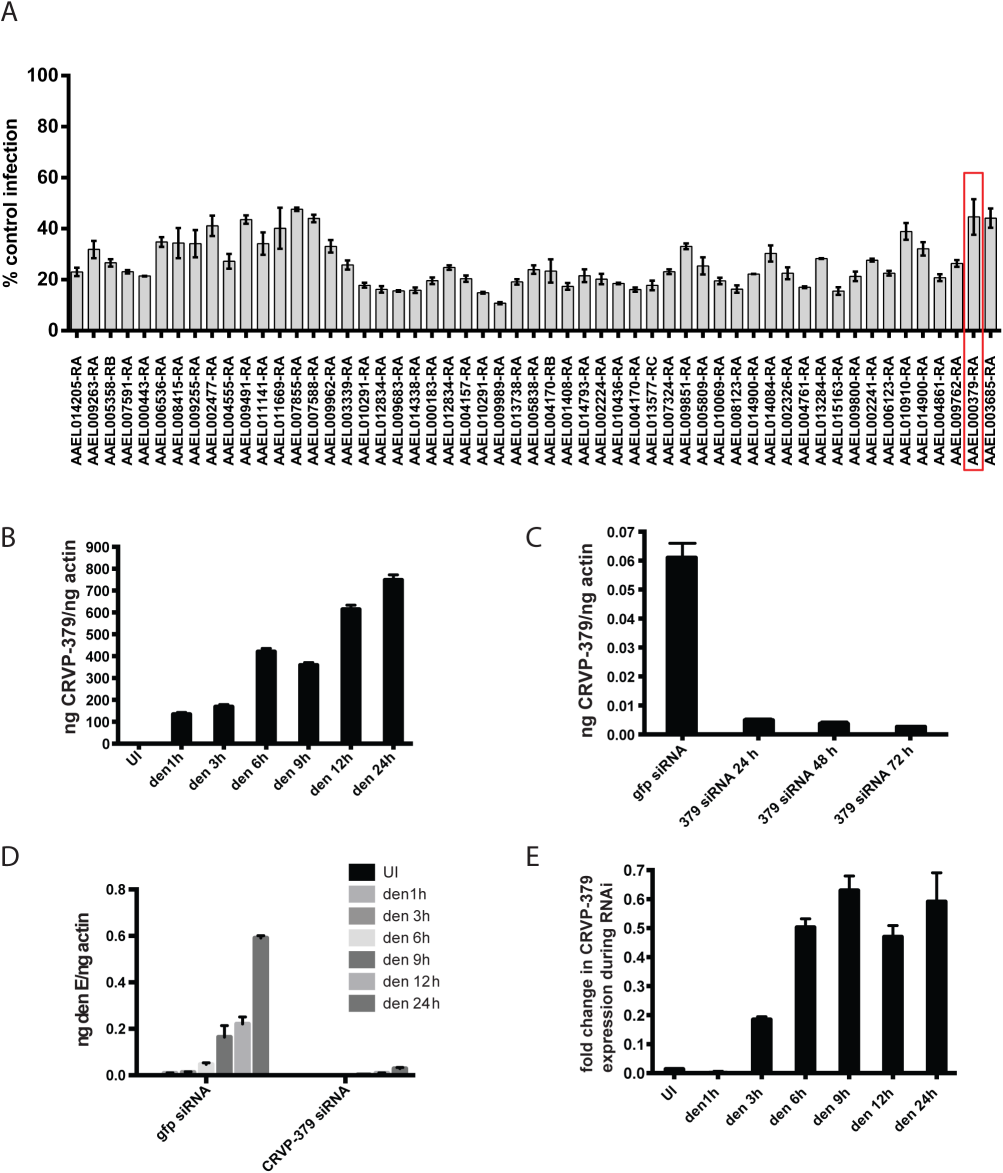
Silencing select virally-up-regulated genes reduces DENV infection in mosquito cells. A. The mosquito genes listed in S1 Fig were knocked down in Aag2 cells using RNAi and the effects on DENV infection were analyzed. The genes that reduced infection below 60% of control are shown. Aag2 cells were infected with DENV (MOI of 1.0) 72 h post-knockdown and analyzed for infection by qRT-PCR 24h post-infection. Data is displayed as percent control infection (using scrambled siRNA). Both DENV infection and qRT-PCR analysis were done in triplicate, data is pooled and error bars indicate standard deviation. B. DENV infection increases CRVP379 in Aag2 cells over time. Aag2 cells were infected with DENV (MOI of 1.0) and infection was measured using qRT-PCR analysis at the timepoints indicated. P<0.01. C. Expression of CRVP379 during RNAi knockdown. CRVP379 siRNA was transfected into Aag2 cells and gene expression was analyzed by qRT-PCR. Samples were taken at 24, 48 and 72h post-knockdown. Expression after transfection of GFP control siRNA is also indicated. D. Reduction of CRVP379 reduces DENV infection over time. Either siRNA against CRVP379 or GFP was transfected into Aag2 cells and cells were infected with DENV (MOI of 1.0) at 72 h post-knockdown. Cells were analyzed for infection by qRT-PCR at the timepoints indicated. E. DENV infection increases CRVP379 expression during siRNA knockdown. Either siRNA against CRVP379 or GFP was transfected into Aag2 cells and cells were infected with DENV at 72 h post-knockdown. Gene expression was analyzed by qRT-PCR at the timepoints indicated. Data is expressed as the fold change in CRVP379 expression in cells with GFP siRNA versus cells with CRVP379 siRNA during DENV infection. B-E. Data is pooled from 6 separate experiments, error bars indicate standard deviation.

We previously used microarray analysis to identify a number of *Ae*. *aegypti* genes that were significantly up-regulated during infection with DENV and other selected flaviviruses [35]. These genes are likely required for flaviviral infection of *Ae*. *aegypti* or are part of the mosquito immune response to viral infection. To elucidate the role of these genes and their corresponding proteins, we reduced gene expression through RNAi knockdown and analyzed the effect on viral infection. We designed siRNA against 100 genes that were significantly up-regulated during DENV infection of *Ae*. *aegypti* (S1 Fig). The siRNA was used to silence these genes in an *Ae*. *aegypti* cell line, Aag2, and the resulting effects on DENV infection were examined. Cells were infected with DENV 72h after siRNA transfection and analyzed for infection using qRT-PCR 24h post-infection. We found that gene silencing both increased and decreased DENV infection, as expected (S2 Fig). The silencing of 9 genes caused cytotoxicity beyond our ability to accurately measure infection levels. Silencing approximately 55 individual genes decreased DENV infection of the cells to below 60% of control infection (Fig 1A), which is greater than 40% inhibition of infection. A number of these genes encode hypothetical proteins for which the function is not known. Several of our target genes do have putative known functions, including a cytochrome P450 (AAEL009762), histone H3 (AAEL003685) and a cysteine-rich venom protein (AAEL000379).

The cysteine-rich venom protein (CRVP), which we will call CRVP379, was a particularly interesting target. CRVP proteins are known to contain a trypsin inhibitor-like (TIL) domain, which indicates that the CRVP379 protein could be a serine protease inhibitor. Both serine proteases and their inhibitors are known to be involved in DENV infection and pathogenesis in the mosquito vector as well as in mammals [50–55]. The expression levels of 19 mosquito CRVP genes were examined during flavivirus infection with the previous microarray analysis. CRVP379 was the only CRVP significantly up-regulated in *Ae*. *aegypti* during infection with any of the 3 prototypic flavivirus infections, including DENV, West Nile virus (WNV) and Yellow Fever virus (YFV), at all timepoints tested (Table 1) [35]. In addition, the 19 CRVP genes that we looked at in our microarray actually have very low sequence identify at the amino acid level (S3 Fig). This indicates that they may not have identical or even similar functions, though they are grouped in a protein family due to the presence of multiple cysteines and a TIL domain. Therefore, we decided to assess the role of CRVP379 in DENV infection of the mosquito vector in greater detail.

**Table 1:** Expression of CRVPs in *Ae*. *aegypti* during flavivirus infection.

SeqID	YFV	DENV2	WNV
AAEL000379	7.4, 6.6, 7.4	5.2, 3.1, 3.0	3.9, 3.1, 1.7
AAEL000353	-2.5, -4.4, -4.2	1.1, 1.5, 1.9	-1.5, -2.6, 1.2
AAEL005090	2.3, 4.3, 2.9	1.2, -1.4, 1.2	2.5, 1.2, 1.5
AAEL000363	1.1, -1.6, -2.3	1.3, 1.0, 1.9	-1.4, -1.2, 1.0
AAEL000317	1.2, -2.6, -4.7	1.4, 1.0, 1.9	-1.1, -2.1, -1.0
AAEL000369	-1.5, -1.7, -1.7	-1.0, 1.1, 2.5	-1.2, -1.6, 1.1
AAEL000333	-1.4, -4.1, -6.7	1.3, 1.1, 1.9	-1.4, -2.3, -1.1
AAEL000302	1.1, -2.2, -1.5	1.3, 2.3, 3.9	-1.2, -1.8, 1.2
AAEL000356	-2.6, -3.2, -2.4	-1.1, 1.0, 2.4	-1.7, -2.7, -1.2
AAEL002667	1.0, 2.1, 1.6	-1.6, 1.0, 1.0	-2.4, 1.9, 3.2
AAEL000361	1.6, -2.2, -1.5	1.9, 1.5, 1.0	1.1, -1.1, -1.3
AAEL005098	1.2, -1.1, -1.3	1.3, 1.0, 1.2	1.4, -1.3, -1.4
AAEL000351	-1.6, -2.3, -2.5	1.2, -1.1, 1.1	-1.1, -1.6, -1.2
AAEL000375	-2.2, -3.1, -1.5	1.7, 1.1, 2.3	-1.9, -2.3, 1.1
AAEL000374	-1.0, -1.8, -3.3	-2.2, -3.3, -1.8	-1.8, -2.5, -2.3
AAEL012194	-1.2, -1.6, -3.2	-2.5, -2.8, -1.6	-1.9, -2.4, -1.9
AAEL005487	1.5, 2.5, 2.8	1.3, 1.5, 1.4	2.2, 2.4, 1.5
AAEL013122	1.6, 1.3, 1.7	1.2, -1.2, -1.1	-1.1, 1.4, 1.5
AAEL000323	-1.2, -2.8, -1.2	1.1, 1.1, 1.2	1.5, 1.1, 2.5

Values are expressed as fold-change over mock infection at time points Day 1, 2, 7.

### DENV infection requires CRVP379 in mosquito cells

Looking at gene expression over time in DENV-infected Aag2 cells, we found that CRVP379 expression increased more than 800-fold when compared to mock-infected cells (Fig 1B, P<0.01). Since the gene was upregulated in mosquito cells during DENV infection, we wanted to examine the phenotype during DENV infection with loss of function experiments. We used RNA interference (RNAi) with siRNA to reduce CRVP379 gene expression. To confirm gene knockdown, levels of CRVP379 were measured after siRNA transfection over time (Fig 1C), and the expression levels remained below 10% at 72h post-transfection. To determine how the reduction of CRVP379 altered DENV infection, we examined infection levels in Aag2 cells at various timepoints from 1 to 24h post-infection during siRNA knockdown. Silencing CRVP379 reduced DENV infection at all timepoints measured, as compared to infection in control cells transfected with siRNA against GFP (Fig 1D). Interestingly, we noticed that levels of CRVP379 were slightly elevated during DENV infection even during siRNA knockdown, when compared to uninfected cells. This can be seen by looking at the fold change in CRVP379 expression in GFP siRNA-transfected cells as compared to CRVP379 siRNA-transfected cells during DENV infection over time (Fig 1E). Together, these results indicated that the silencing effects of RNAi on CRVP379 are slightly overcome by the gene upregulation during DENV infection, but that infection levels still remained quite low when compared to cells with no CRVP379 silencing.

### Endogenous CRVP379 is sufficient for optimal DENV infection

**Fig 2:**
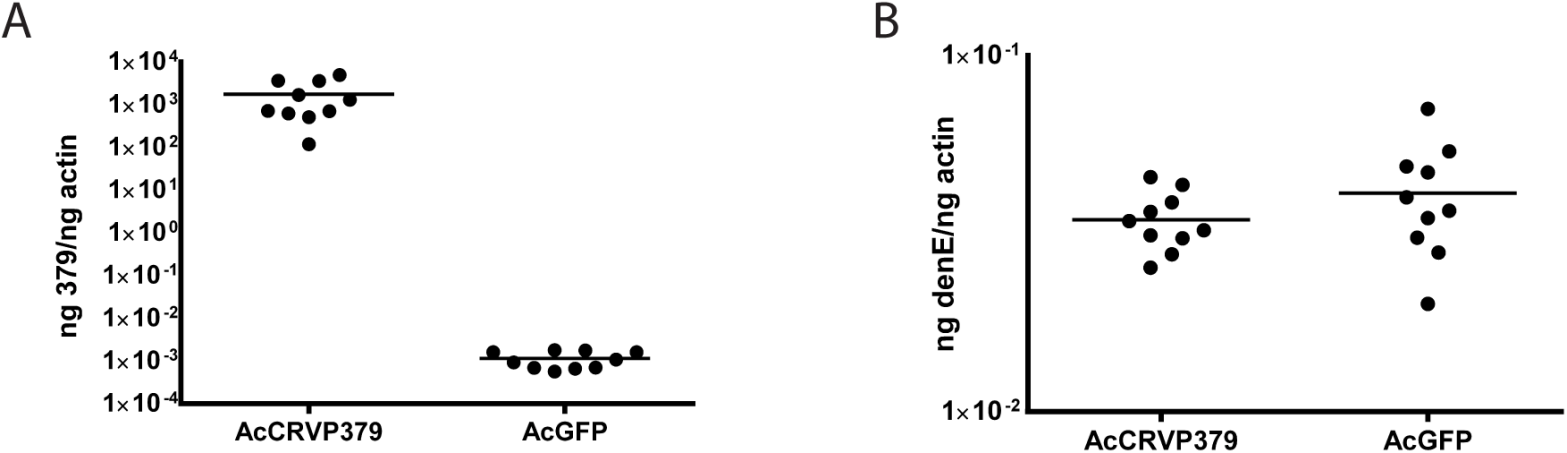
DENV infection optimally enhances CRVP379 expression. Aag2 cells were transfected with an insect expression vector encoding CRVP379 (AcCRVP379) or GFP (AcGFP) and A. CRVP379 expression was measured by qRT-PCR at 48 h post-transfection. B. Cells were infected with DENV (MOI of 1.0) at 48 h post-transfection and infection levels were measure by qRT-PCR at 24 hpi.

Since we found that the presence of CRVP379 is required for DENV infection, we wanted to test whether increasing CRVP379 levels would enhance infection levels. To do this, we cloned the CRVP379 coding region into the insect expression vector pAc5.1/V5-His (Life Technologies, CA), resulting in pAcCRVP379 vector. This expression vector was transfected into an *Ae*. *aegypti* mosquito cell line, Aag2, and the cells were subsequently infected with DENV. A vector expressing GFP was transfected as a control into a separate group of DENV-infected cells. Transfection levels as measured by GFP transfection are over 50%, which will give meaningful results when looking at gene expression and effects on DENV infection (S4 Fig). At 48h post-infection, levels of CRVP379 expression were measured by qRT-PCR. The expression levels of CRVP379 were over 1000 times higher in the cells that were transfected with the CRVP379 plasmid (Fig 2A). We next infected the transfected cells with DENV at 48h post-transfection. At 24 hpi, RNA was isolated and qRT-PCR done to measure DENV infection in the cells. We found that the overexpression of CRVP379 did not increase DENV infection levels in the cells (Fig 2B). This indicated that the endogenous levels of CRVP379 protein are sufficient and that the virus has likely evolved to require only those amounts for optimum infection.

### Acquisition of DENV requires CRVP379 in live mosquitoes

**Fig 3:**
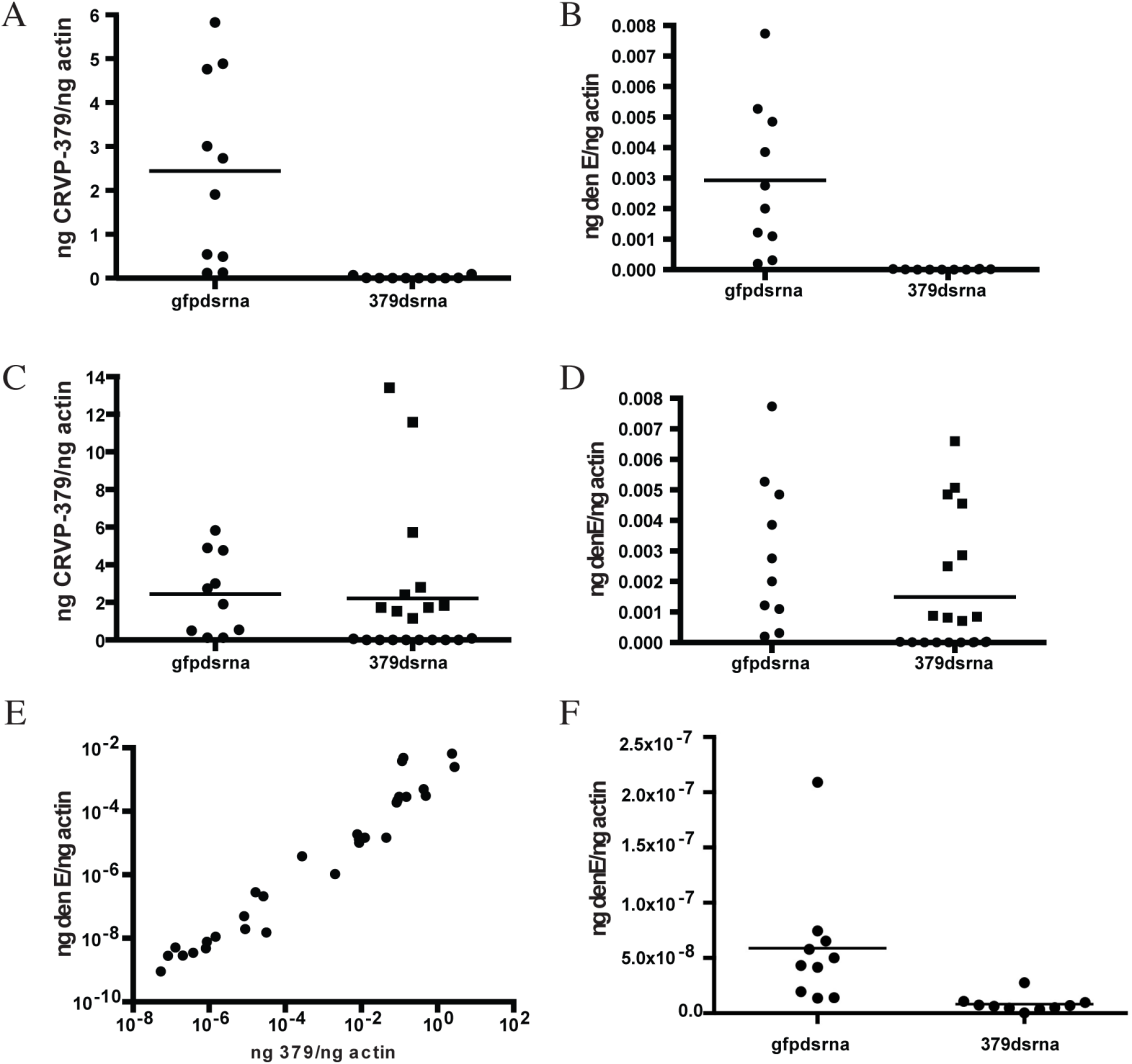
Silencing CRVP379 inhibits DENV acquisition in live mosquitoes. A-E. Mosquitoes were intra-thoracically injected with either dsRNA against the coding region of CRVP379 or dsRNA against GFP as control. At 4 dpmi, mosquitoes were infected with DENV through blood feeding. At 4 dpi, midgut tissues were dissected and individually analyzed for gene expression with qRT-PCR analysis. Each data point represents one mosquito midgut. A. Levels of CRVP379 in select midguts where RNAi was successful, as compared to levels in control mosquitoes. P<0.01. B. Levels of DENV infection in select midguts where RNAi was successful, as compared to levels in control mosquitoes. P<0.01. Infection rates in midguts where CRVP379 RNAi was successful ranged from to .000000765–.0000315 ng DENV E/ng actin. C. Levels of CRVP379 in midguts where RNAi was both successful and unsuccessful, as compared to levels in control mosquitoes. D. Levels of DENV infection in midguts where RNAi was both successful and unsuccessful, as compared to levels in control mosquitoes. C-D. Squares represent midguts where RNAi did not knock down CRVP379 successfully, cirlces represent midguts where RNAi did knock down CRVP379 successfully. E. Levels of DENV infection correspond to levels of CRVP379 expression. Both midguts where RNAi did and did not knock down CRVP379 were analyzed for both CRVP379 expression and DENV infection by qRT-PCR. Data is plotted as ngs of DENV E versus levels of CRVP379, normalized to mosquito actin. Data correlated with Pearson, r = 0.6442, P<0.0001 F. Silencing CRVP379 reduces DENV infection in whole mosquitoes. Mosquitoes were intra-thoracically injected with either dsRNA against the coding region of CRVP379 or dsRNA against GFP as control. At 4 dpmi, mosquitoes were infected with DENV through blood feeding. At 7 dpi, homogenized whole mosquitoes were individually analyzed for gene expression with qRT-PCR analysis.

Since we found that CRVP379 was required for DENV infection of mosquito cells, we decided to next look at the requirements for infection in live *Ae*. *aegypti*. To do this, we designed dsRNA against the CRVP379 coding region and inoculated mosquitoes via intra-thoracic injection. At days 2, 4 and 8 post-injection, we dissected out midgut tissues and measured levels of CRVP379 expression by qRT-PCR analysis. Although knockdown was not achieved in all tissues tested, a near-complete reduction of CRVP379 expression (over 95%) in midguts was seen 70% of the time by day 8 (Fig 3-all panels). We next examined the effects of silencing CRVP379 on DENV acquisition in the mosquito midgut. Mosquitoes were again injected with dsRNA against CRVP379 or a control dsRNA against GFP protein. At day 4 post-injection, mosquitoes were infected with DENV by blood feeding using a hemotek apparatus. At day 4 post-infection, midgut tissues were dissected out and analyzed for both CRVP379 expression and DENV infection by qRT-PCR. Since not all midgut tissues had reduction in CRVP379 expression, we analyzed each midgut individually in order to examine the effects on DENV infection in midguts that did have reduced CRVP379. The levels of CRVP379 in the selected midguts are shown in Fig 3A. In the midguts that had reduced CRVP379 expression, DENV infection was almost completely inhibited, as compared to infection in control mosquito midguts (Fig 3B, P<0.01). We also analyzed the data after adding back in the midguts that did not have sufficient gene knock down and looked at levels of DENV infection. Fig 3C shows the levels of CRVP379 in these midguts. Interestingly, in midgut tissues where CRVP379 was not knocked down, DENV infection was comparable to levels in the GFP dsRNA-injected mosquitoes (Fig 3D-squares). Plotting the data points as level of DENV versus level of CRVP379, there is a correlation between expression of CRVP379 in the mosquito midgut and level of DENV infection in that same midgut (Fig 3E, r = 0.6442, P<0.0001). This indicates that CRVP379 levels are directly related to levels of DENV infection in the mosquito midgut. Finally, we repeated the RNAi experiment and allowed the DENV infection to disseminate for 7 days. At day 7 post-infection, whole mosquitoes were homogenized and analyzed for both CRVP379 expression and DENV infection by qRT-PCR (Fig 3F). The mosquitoes that received the dsRNA against CRVP379 had a significant reduction in DENV infection levels as compared to the control mosquitoes. This indicates that the reduction of CRVP379 blocks DENV infection in the whole mosquito.

### During DENV infection, CRVP379 interacts with mosquito prohibitin, a putative DENV receptor

After establishing that DENV requires CRVP379 in both mosquito cells and live *Ae*. *aegypti*, we next wanted to investigate the mechanistic role that CRVP379 plays during infection. To do this, we used the tandem affinity purification (TAP) assay to identify putative mosquito proteins that bind CRVP379 during DENV infection. We cloned the coding region of CRVP379 into the NTAP vector (Stratagene, CA), which fuses the gene to purification tags, and transfected this plasmid into Aag2 mosquito cells. Cells were infected with DENV 24h post-transfection and lysed 24h post-infection. The cell lysate was processed and CRVP379 was purified using the expressed tags, along with interacting mosquito proteins. The resulting solution was sent for LC/MS-MS analysis to determine which proteins were pulled out of the mosquito cell lysate by CRVP379 during DENV infection. A separate set of cells was transfected with the NTAP vector expressing GFP as control. Table 2 lists the mosquito proteins that putatively bound CRVP379 and were not identified during control experiments. One of the proteins identified, prohibitin, was previously characterized as binding to DENV in *Aedes* A7 cells [56] and has also been suggested as a putative DENV receptor in mosquito cells [57]. Since we found that prohibitin binds CRVP379 during DENV infection using the TAP assay, this may indicate that the proteins act together to facilitate DENV entry into mosquito cells.

**Table 2:** CRVP379 tandem affinity purification assay results.

SeqID	Gene	Score	Expectation
AAEL012282	prohibitin	839	2.10E-80
AAEL003303	hypothetical	212	9.70E-18
AAEL008103	40S ribosomal	450	1.80E-41
AAEL007771	60S ribosomal	353	7.90E-32
AAEL011089	ribonucleoprotein	980	1.80E-94
AAEL001471	hypothetical	357	3.60E-32
AAEL006158	histone H3	152	9.80E-12
AAEL003415	lamin	611	1.30E-57
AAEL011197	actin	1458	0.00E+00
AAEL009883	26S proteasome subunit	276	4.00E-24
AAEL000482	histone H3	214	6.40E-18
AAEL006642	tubulin alpha chain	1350	0
AAEL007609	histone H2A	242	9.60E-21
AAEL008517	elongation factor tu	922	1.10E-88
AAEL015674	histone H2B	913	8.90E-88
AAEL010668	quinone oxioreductase	1276	4.10E-124
AAEL013739	electron transport oxioreductase	372	1.00E-33
AAEL010146	3_H-Coa dehydrogenase	608	2.50E-57
AAEL003530	ribosomal protein P1	288	2.80E-25
AAEL011180	hypothetical	250	1.90E-21
AAEL012736	ribosomal protein L15	344	6.70E-31
AAEL004317	hypothetical	212	1.10E-17
AAEL006511	ubiquitin	213	8.10E-18
AAEL008103	40S ribosomal	450	1.80E-41
AAEL015464	histone H1	339	2.10E-30
AAEL011584	chaperonin 60kD	743	8.60E-71
AAEL010608	succinate dehydrogenase	173	9.10E-14
AAEL004325	ribosomal L5	362	1.10E-32
AAEL008188	60S ribosomal	189	2.00E-15

**Fig 4:**
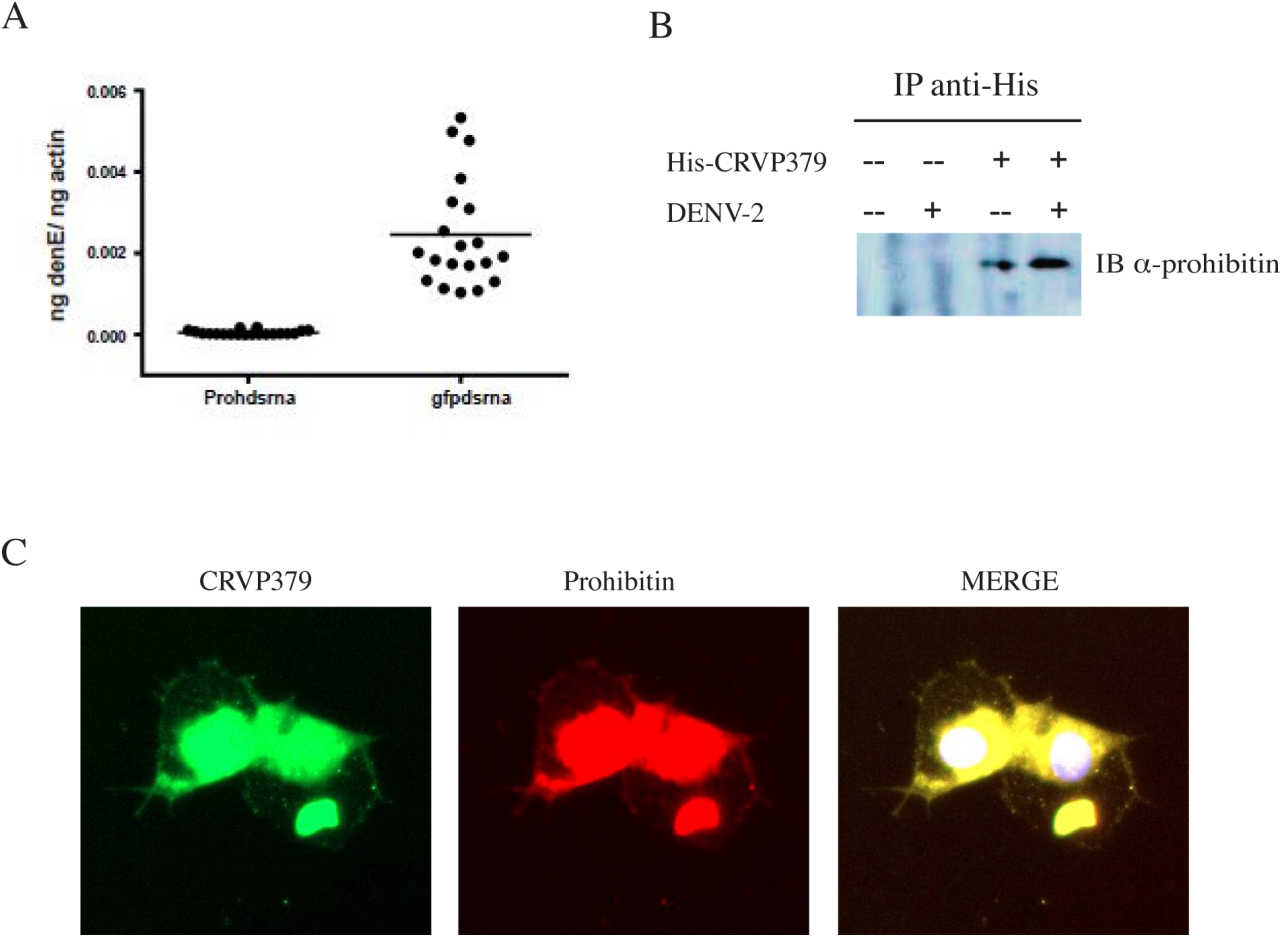
CRVP379 interacts with mosquito prohibitin during DENV infection. A. Prohibitin is required for DENV infection of *Aedes aegypti*. Mosquitoes were intra-thoracically injected with either dsRNA against the coding region of prohibitin (Prohdsrna) or dsRNA against GFP (gfpdsrna) as control. At 4 dpmi, mosquitoes were infected with DENV through blood feeding. At 4 dpi, midgut tissues were dissected and individually analyzed for gene expression with qRT-PCR analysis. Each data point represents one mosquito midgut. P<0.01. B. Aag2 cells were transfected with His-tagged CRVP379 and/or infected with DENV. At 48h post-infection, antibodies against the His tag were used to pull the His-tagged CRVP379 protein out of cell lysates. The precipitated solution was run on SDS-PAGE gel and Western blot analysis was performed using antibody against prohibitin. C. CRVP379 and prohibitin co-localize in mosquito cells during DENV infection. Aag2 cells were transfected with His-tagged CRVP379 and infected with DENV 48h post-transfection. Cells were fixed in 4% paraformaldehyde 24h post-infection and stained with antibodies against the His-tag (green) and against prohibitin (red). DAPI was used to localize the nucleus. Representative images are shown.

In establishing prohibitin as a putative receptor in mosquito cells, Kuadkitkan et al used siRNA against the mosquito prohibitin gene to inhibit protein production and saw a significant decrease in DENV infection in mosquito cells [57]. To confirm that prohibitin is required for DENV infection in mosquitoes, we designed dsRNA against the mosquito prohibitin gene and examined the impact of silencing prohibitin on DENV infection. *Ae*. *aegypti* were intra-thoracically inoculated with the dsRNA and at 4 days post micro-injection (dpmi), mosquitoes were infected with DENV via blood feeding. At 4 days post-infection (dpi), mosquito midguts (MG) were dissected and analyzed for infection by qRT-PCR analysis. Our results show that DENV infections levels were greatly inhibited by prohibitin silencing as compared to the control mosquitoes (Fig 4A), confirming a requirement for prohibitin in DENV mosquito infection. We next performed co-immunoprecipitation to confirm the protein interaction between CRVP379 and prohibitin. Aag2 cells were transfected with an expression vector coding for His-tagged CRVP379 protein and/or infected with DENV. Cells were then lysed and antibody against the His tag was used to pull down His-CRVP379 from the cell lysate. Western blot analysis identified prohibitin protein in the immunoprecipitate (Fig 4B), demonstrating that His-tagged CRVP379 bound prohibitin during DENV infection in the mosquito cells. We then used immunoflourescent imaging to visualize the putative prohibitin-CRVP379 protein interaction. Aag2 cells were transfected with the His-tagged CRVP379 expression construct and infected with DENV 48 hours post-transfection. At 24 hours post-infection, cells were fixed and stained with antibodies against the His tag and prohibitin protein. Fig 4C shows that the two proteins were highly colocalized during DENV infection. We next wondered whether prohibitin overexpression could rescue the mosquito cells that were resistant to DENV infection due to reduced CRVP379 expression. To investigate this, we transfected CRVP379 siRNA into Aag2 cells and then overexpressed mosquito prohitibin before infecting the cells with DENV. We found that the overexpression of prohibitin did not significantly increase DENV infection in cells with reduced CRVP379, though there was a slight enhancement (S5 Fig). This indicates that, though the proteins may act together to facilitate DENV infection in mosquito cells, prohibitin cannot replace the function of CRVP379 protein. Interestingly, the overexpression of prohibition in control Aag2 cells (with siRNA against GFP) did increase DENV infection (S5 Fig).

### Antisera against CRVP379 inhibits DENV infection in mosquitoes

**Fig 5:**
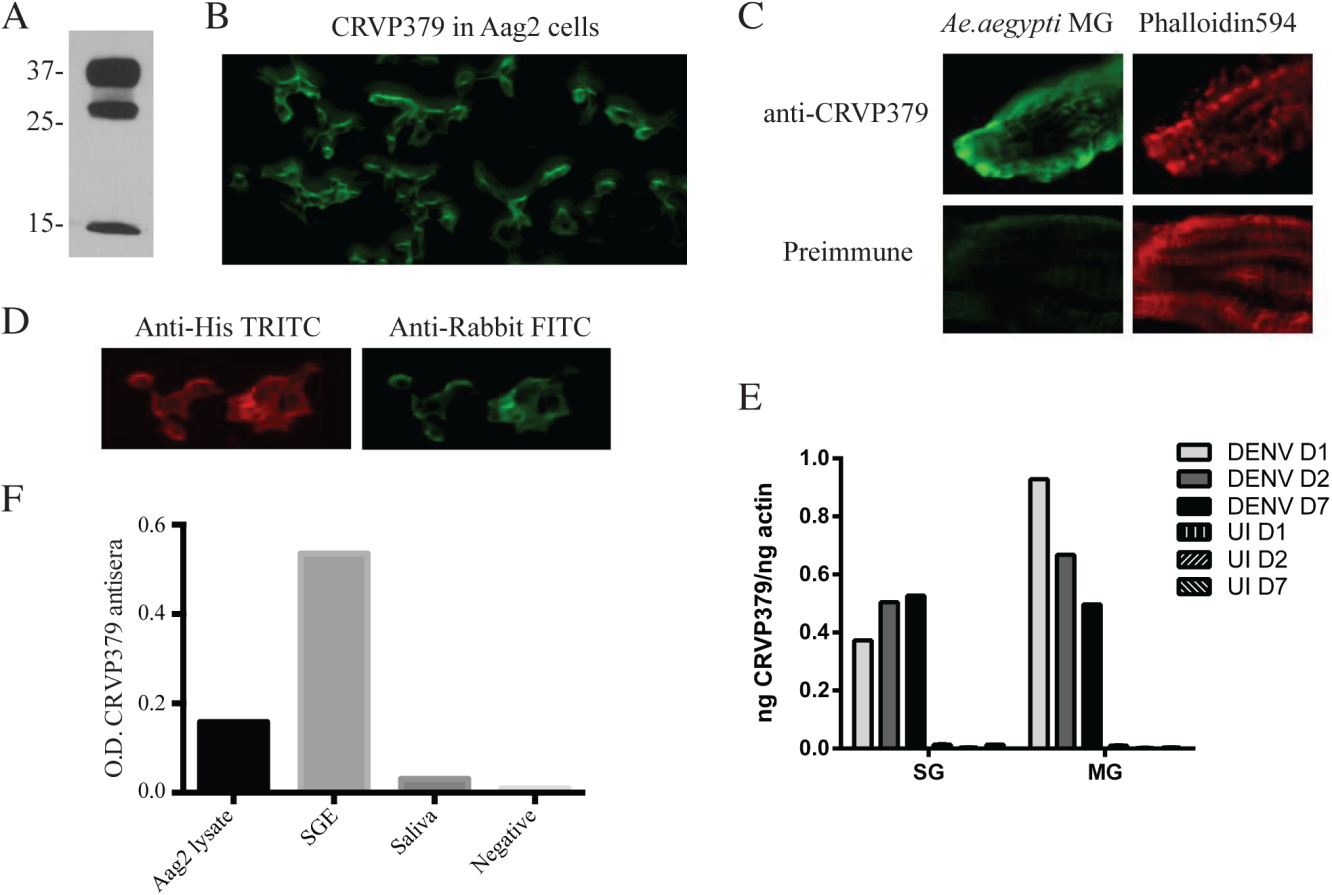
Antibodies against CRVP379 recognize native protein in *Ae*. *aegypti*. A. CRVP379 antisera binds recombinant protein. An SDS-PAGE gel was run using rCRVP379 and Western blot analysis was done using the CRVP379 antisera. B. Antibodies were made against mosquito CRVP379 and used to bind endogenous protein in the Aag2 cell line. Aag2 cells were infected with DENV and cell fixed 24 hpi for staining. A representative image is shown at 20X. C. CRVP379 antibodies bind endogenous CRVP379 in mosquito midguts. *Ae*. *aegypti* MG were dissected and fixed in 4% paraformaldehyde before staining. Both preimmune sera and sera from CRVP-379 injected mice were used for staining (green). Phalloidin594 was used to highlight midgut structure (red). D. Antisera against CRVP379 recognizes the CRVP379 protein in mosquito cells. Aag2 cells were transfected with an expression construct encoding His-tagged CRVP379 protein. Cells were fixed 48h post-transfection and stained with both CRVP379 antisera and antibody against the His tag. Secondary antibodies were used as indicated. E. CRVP379 is increased in both MG and SG of DENV-infected mosquitoes. Mosquitoes were either infected with DENV or mock solution and organs dissected at 1, 2 and 7 dpi. CRVP379 gene expression was analyzed by qRT-PCR analysis and is shown as ng CRVP379 normalized to actin. DENV was used at 10^5^ PFU/mL for infection in mosquitoes. F. CRVP379 antisera binds mosquito cells and tissues. ELISA analysis was done with Aag2 cell lysate, *Aedes aegypti* salivary gland extract (SGE) and *Aedes aegypti* extracted saliva. O.D. values at 450nm are presented on the graph.

**Fig 6:**
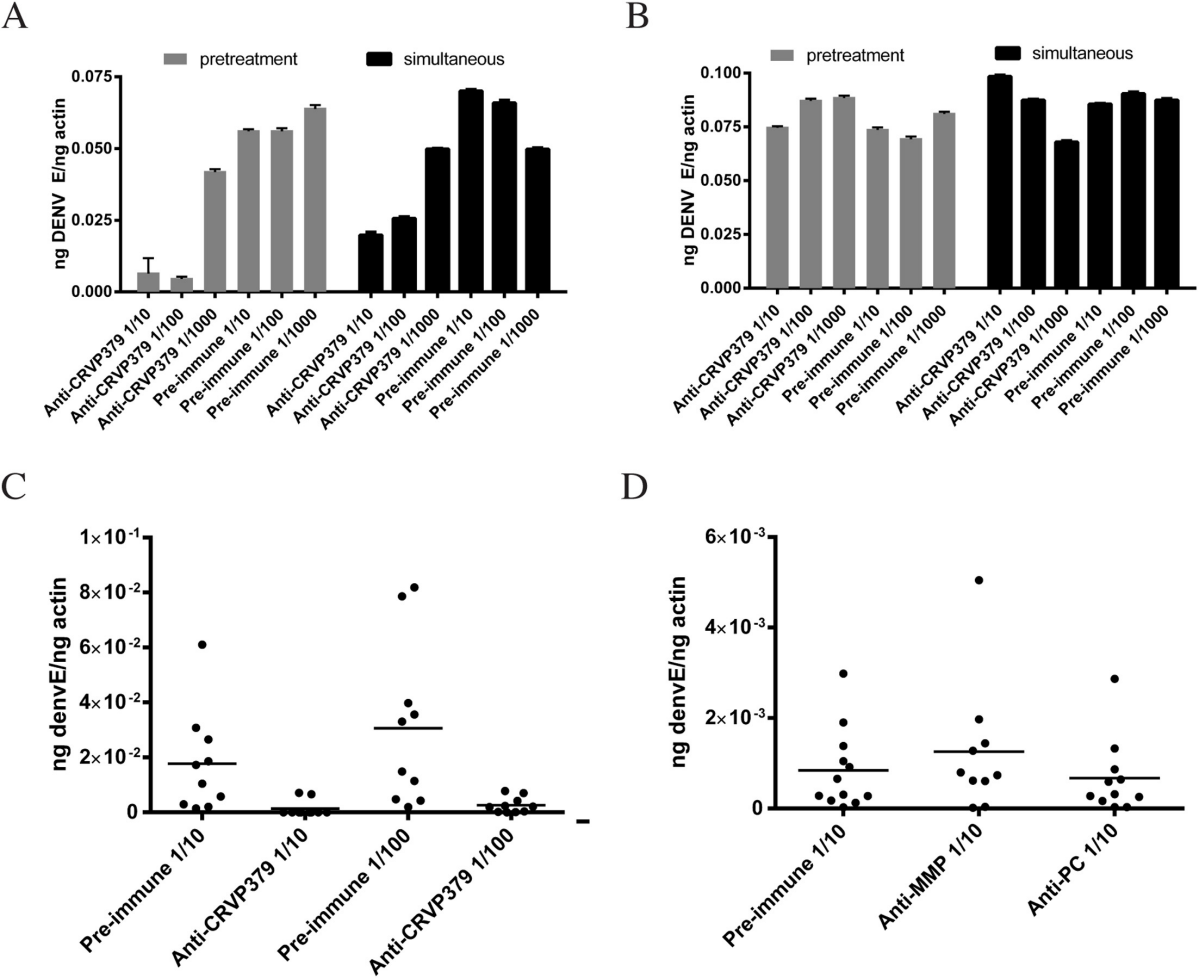
CRVP379 antisera blocks infection of DENV in mosquitoes. A/B. CRVP379 antisera inhibits DENV infection in mosquito cells. Aag2 (A) or Huh7 (B) cells were either incubated with antisera against CRVP379 or control preimmune sera for 2h at RT and then infected with DENV (pretreatment group) or antisera against CRVP379 or control preimmune sera was incubated with DENV for 1h at RT and then added to cells (simultaneous group). Infection was analyzed by qRT-PCR at 24 hpi. C/D. CRVP379 antisera inhibits DENV infection in mosquitoes. *Ae*. *aegypti* were fed a mixture of blood, DENV and either CRVP379 antisera or preimmune sera as indicated. Antisera were used at dilutions of 1/10 or 1/100 (C). Infection rates in midguts with CRVP379 antisera ranged from to .00008785–.07833 ng DENV E/ng actin. A separate group of mosquitoes was fed antisera against control mosquito proteins, MMP and PC (D). At 3 dpi, mosquito MG were dissected and qRT-PCR analysis done to quantify DENV infection. Results are shown as ng DENV E normalized to mosquito actin. Each data point represents one MG.

Since we found that inhibiting CRVP379 gene expression using RNA interference reduced DENV in *Ae*. *aegypti*, we next wanted to try and inhibit protein function with antibody and examine the effects on DENV infection. To do this, recombinant protein consisting of residues 21–128 of CRVP379 was expressed in *E*. *coli* along with a GST tag for purification. To generate polyclonal antiserum, rabbits were immunized with the recombinant CRVP379 (rCRVP379). We used the antisera in Western blot analysis to confirm that antibodies would bind the recombinant protein (Fig 5A). We next ensured that the polyclonal antisera contained antibodies that recognized endogenous CRVP379 protein in the mosquito. To test this, we used the antisera to stain Aag2 cells and found that there was a strong reaction between the CRVP379 antisera and protein in the cells (Fig 5B). We then used the antisera to probe mosquito midgut tissue for endogenous protein. Fig 5C demonstrates that the CRVP379 antisera, but not the pre-immune control sera, recognized protein in the dissected mosquito MG tissue. We also used the antisera to probe MG tissue with reduced CRVP379 expression due to RNAi (S6 Fig). To confirm that the antisera did recognize the CRVP379 protein in the mosquito, we ectopically expressed a His-tagged CRVP379 protein in Aag2 cells and used antibody against the His tag along with the CRVP379 antisera. Staining with the CRVP379 antisera colocalized with the anti-His staining, indicating that the antisera recognized the CRVP379 protein (Fig 5D). We then looked at tissue-specific expression of CRVP379 and found that levels are increased in both the salivary glands (SG) and midguts (MG) of DENV-infected mosquitoes, as compared to uninfected mosquito tissues, at all timepoints examined (Fig 5E). We also used ELISA analysis with the CRVP379 antisera using both Aag2 cell lysate, *Ae*. *aegypti* salivary gland tissue and *Ae*. *aegypti* saliva to confirm that the antisera bound endogenous CRVP379 protein (Fig 5F). Next, we tested the effects of the antisera on DENV infection in Aag2 cells. We used two experimental protocols; in one, the antisera was incubated with the cells for 2h at RT and then infected with DENV (pretreatment group), in the second, antisera and DENV were incubated for 1h at RT and then added to cells (simultaneous group). We used pre-immune sera for a control and also did the same experiment in the Huh-7 human liver cell line as an additional control, as antisera against a mosquito protein should not have an effect on DENV infection in mammalian cells. Infection was analyzed by qRT-PCR analysis at 24 hpi. We found that the antisera against CRVP379 inhibited DENV infection in Aag2 cells at dilutions up to 1/100 (Fig 6A). We also found that incubating the antisera with the cells before DENV infection resulted in a slightly larger reduction in infection levels (Fig 6A). We did not see any reduction in DENV infection in either experimental group using Huh-7 cells (Fig 6B). We then tested the effects of the antisera against CRVP379 on DENV infection in *Ae*. *aegypti*. Mosquitoes were fed a mixture of human blood, DENV and either CRVP379 antisera or preimmune sera at 1/10 and 1/100 dilutions. We also used control antisera against two unrelated, GST-tagged mosquito proteins MMP (AAEL003012) and PC (AAEL011045). On 3 dpi, mosquito MG were dissected and qRT-PCR was done to analyze DENV infection. The antisera against CRVP379 significantly reduced the DENV infection in the mosquitoes at both 1/10 and 1/100 dilution as compared to mosquitoes which fed on the preimmune sera (Fig 6C). The antisera against the control GST-tagged proteins did not reduce DENV acquisition in the mosquito MG tested (Fig 6D).

## Discussion

Flaviviruses are known to modify gene expression in their mosquito transmission vectors during infection. Our previous results showed that infection of *Ae*. *aegypti* with either DENV, WNV or YFV, modifies expression levels of at least 405 genes [35]. The study of mosquito genes modified during flavivirus infection may lead to the identification of key vector antiviral mechanisms as well as key factors for interruption of the viral life cycle. One of the genes that we identified as being significantly upregulated during DENV infection was the CRVP379 gene. Cysteine-rich venom protein (CRVPs) are members of a large family of cysteine-rich secretory proteins (CRISPs), predominantly found in mammalian males and reptile venom [58]. CRISPs contain characteristic cysteine rich C-terminal domains thought to act as ion channel regulators [58] and are also characterized by their role in proteolytic and defense mechanisms [59]. CRISPs and CRVPs have been described in a broad spectrum of insects and higher vertebrates [59–62].

Recently, Bonizzoni et al found several CRVP genes to have differentially regulated expression during DENV infection of *Ae*. *aegypti*, including CRVP379, which was shown to be upregulated on day 1 and day 14 in the mosquito MG during infection [42]. Here, we found that CRVP379 was the only CRVP significantly upregulated in mosquitoes after infection with DENV. Furthermore, knockdown of CRVP379 protein both *in vivo* and *in vitro* was able to reduce viral infection, and we found a significant positive association between the level of CRVP and DENV infection. This indicates that CRVP379 is specifically required for DENV infection of *Ae*. *aegypti*, at least in our current studies. The genetic variations between both DENV and mosquito strains likely contributes to differences among various studies, and consistent reporting of these discrepancies warrants further research into these variations along with additional transcriptomic analysis of the impact of DENV infection on mosquitoes.

Recent work has shown that another mosquito venom protein, a member of the antigen-5 family (Ag5), is upregulated in the salivary glands of *Ae*. *aegypti* during Chikungunya virus infection [63]. This protein is present in the saliva of several insects and is associated with platelet aggregation inhibition in blood sucking arthropods [46,64]. More investigation is needed to determine whether mosquito CRVP proteins and the Ag5 proteins are related or have similar functions during virus infection. Another group of CRVP proteins, the Cysteine-rich secretory proteins, Antigen 5, and Pathogenesis-related 1 proteins (CAP) superfamily, has been descried in the sialotranscriptome of *Ae*. *Aegypti* as well as in *Culex* [49,65,66]. Previous reports indicate that these genes could be preferentially expressed in the salivary glands of female mosquitos, perhaps suggesting an important role during blood feeding. Our study showed that up-regulation of CRVP379 occurs in both midgut and salivary glands of mosquitoes, and expression in salivary glands increased from day 1 to day 7 during DENV infection. This suggests that CRVP379 may be found in the saliva of DENV-infected mosquitoes. We have previously found that *Ae*. *aegypti* and certain *Anopheles* saliva have the ability to induce an antibody response in humans that can be correlated with the level of exposure to mosquito bites and disease status [67,68]. Several insect proteins from the CAP superfamily have also been reported to stimulate mammalian immune responses [69]. Given that CRVP379 has no homologous proteins in humans, we suspect that it will be a potent immunogen if used as a TBV.

Many CRVP proteins contain trypsin inhibitor-like (TIL) domains found in members of the serine protease inhibitor family [70] and functional sequence analysis confirmed that CRVP379 does contain a TIL domain from amino acids 23–79. Serine proteases and their inhibitors are known to have very specific interactions, and they play central roles in many cellular processes [71–73]. In addition, both serine proteases and their inhibitors have been shown to have an impact on DENV infectivity in both mammals and mosquitoes [50,55,74]. As such, we sought to identify the serine protease that CRVP379 potentially bound by using the TAP assay to investigate which mosquito proteins CRVP379 bound during DENV infection. We discovered that CRVP379 interacted with a number of mosquito genes during infection, including histones, ubiquitin and prohibitin.

Previous research has suggested that prohibitin may be a receptor for DENV in mosquitoes, as expression levels of this protein correlate with the susceptibility of DENV infection in both *Ae*. *aegypti* and *Ae*. *albopictus* cell lines [57]. Prohibitin is a protein pervasive expressed and highly conserved in eukaryotic cells [75] and has been previously described as an inhibitor of cell proliferation [76]. Prohibitin is found in several cellular compartments including nucleus, mitochondria and cytoplasm [57]. Furthermore, a recent report shows that Cry4B, one of the major insecticidal toxins produced by *Bacillus thuringiensis israelensis*, co-precipitates and co-localizes with prohibitin in *Ae*. *aegypti* larva midgut, and this interaction is able to reduce DENV infection under physiological conditions [77]. These findings suggest that the inhibition of proteins that interact with viral receptors may potentially block mosquito infection. Additionally, several proteins have been reported to bind prohibitin conferring resistance to bacteria phagocytosis [78] as well as cell surface expressed binding protein [79]. In DENV infection, it has been suggested that prohibitin interacts directly at the cell surface with the viral envelope protein. Our current work shows that CRVP379 is able to interact with several other mosquito proteins including prohibitin, suggesting that CRVP379 may be involved in virus cell entry along with other putative roles.

In spite of decades of effort, there are currently no approved DENV vaccines available. A recent study with a live-attentuated tetravalent DENV vaccine developed by Sanofi Pasteur has demonstrated partial protection against DENV [22] and shows promising results, though efforts continue to develop vaccines that will confer full protection. A vector-based vaccine would nicely complement these efforts at traditional vaccine development and could contribute as an additional strategy to combat the increasing global spread of DENV. The development of vaccines targeting either a pathogen or vector protein to prevent transmission to human hosts is considered essential to the eradication of many emerging tropical diseases, including malaria. Transmission-blocking vaccines (TBVs) are currently being developed and have been shown to be successful at preventing malaria infection of *Anopheles* mosquitoes (12–15). One of these, a TBV developed against the *Plasmodium* protein Pfs25, was able to prevent the transmission of malaria from infected mice to naïve mosquitoes (12,13). Another group found that vaccinating mice with the mosquito protein serpin-2 prevented the transmission of *Plasmodium berghei* to a naïve group of mosquitoes (16). In addition, an arthropod-specific TBV based on the outer surface protein A (OspA) of Borrelia burgdorferi, the causative agent of Lyme disease, has been shown to protect mice from spirochete infection (17). Proteins of the sand fly have also been used successfully as TBVs to prevent the transmission of *Leishmania* (18).

Dengue virus is transmitted to humans in saliva during mosquito probing and blood feeding. During this process, mosquitoes take in host factors contained in the blood including host antibodies, complement proteins and immune cells that remain active for several hours post-feeding [80,81]. Previous studies have shown that the presence of antibodies against mosquito proteins are able to disrupt mosquito infection and transmission of pathogens [82,83]. This type of TBV has several advantages over a TBV targeting pathogen antigens, including the ability to target a conserved molecule among vector genera and that the targeted genes may also affect mosquito survival in nature. Recently, Cheng et al demonstrated that antibodies against mosquito C-type lectin proteins were able to block DENV infection in *Aedes* mosquitoes [34]. Their data strongly suggested that a TBV targeting DENV acquisition in mosquitoes is possible and may be close at hand. Our results inhibiting DENV in *Aedes* using antisera targeting CRVP379 protein showed a similar reduction in viral infection and suggests that CRVP379 may also be a viable target for the development of a TBV. Importantly, CRVP379 has no homolog in humans and extremely low sequence identity to any protein in the human proteome. This means there should not be any off-target immune reaction targeting self if CRVP379 were to be used to stimulate antibody production in humans.

In the present study, we identified a mosquito protein that was required for DENV infection in mosquito cells, CRVP379. We showed a correlation between this protein and DENV infection levels *in vitro* and *in vivo*. The interaction between CRVP379 and prohitibin, a putative viral receptor in mosquitoes, may be the mechanism behind the requirement for infection. In addition, antiserum against CRVP379 protein was able to significantly inhibit DENV acquisition in *Ae*. *aegypti*. Given that the CRVP379 protein was also upregulated in the mosquito during infection with WNV and YFV, it stands to reason that a TBV developed against this protein may act to block acquisition and transmission of multiple, globally important flaviviruses. We have also been able to detect an antibody response against CRVP379 in human serum samples, indicating that the protein is immunogenic. We are currently designing studies to correlate levels of these antibodies with putative protection against dengue virus infection and disease severity upon infection.

## Materials and Methods

### Cell culture and virus growth

The Aag2 *Ae*. *aegypti* cell line (ATCC, VA) was used for transfection and infection studies. The cells were grown at 30°C and 5% CO2 in DMEM supplemented with 10% heat-inactivated fetal bovine serum (Gemini, CA), 1% penicillin-streptomycin and 1% tryptose phosphate broth (Sigma, MO). Dengue virus stock was grown in C6/36 *Ae*. *albopictus* cell line using the same media. The dengue strain used was DENV-2 New Guinea C. Cells were infected at an m.o.i. of 1.0, virus was allowed to propagate for 6–8 days, supernatant was removed, spun down and virus stock was stored at -80°C until use.

### RNAi

S1 Fig provides a complete list of the siRNA molecules used in our *in vitro* knock-down studies. Dharmafect4 reagent (Dharmacon,) was used to transfect the siRNA into the Aag2 cells according to manufacturer’s instrucitons. For gene knockdown in live mosquitoes, dsRNA was produced from 500bp coding regions of either *Ae*. *aegypti* CRVP379, *Ae*. *aegypti* prohibitin or GFP. Briefly, PCR was used to produce a DNA template with T7 overhangs that was then used with the Ambion Megascript kit to produce the dsRNA molecules. The dsRNA was transfected into mosquitoes as described.

### Mosquito infections

The Rockefeller strain of *Ae*. *aegypti* were infected by blood-feeding, using 400 μL of DENV-infected C6/36 cell supernatant added to 1 mL serum-inactivated human donor blood (The Blood Center, New Orleans, LA). Mosquitoes were fed for 20 minutes at room temperature using a hemotek feeder and maintained in groups of 10 at 30°C, 80% humidity. Mosquitoes were supplied sucrose water as a source of dietary sugar. At the conclusion of experiments, mosquitoes were briefly washed in 70% ethanol and then rinsed in sterile PBS. Organs were dissected in sterile PBS and transferred to Eppendorf tubes separately. Mosquito organs were stored in PBS with protease inhibitors for protein assays and homogenized in RLT buffer (Qiagen, CA) for gene expression assays.

### qRT-PCR analysis

RNA was isolated from infected *Ae*. *aegypti* mosquitoes on Days 1, 2, 7 and purified using RNeasy kit (Qiagen, CA) according to manufacturer’s instructions. The quantitative RT-PCR (qRT-PCR) analysis was done using the QuantiFast kit according to manufacturer’s instructions (Qiagen, CA). Oligos for the qRT-PCR reactions were:

DENV envelope: F: 5’-CATTCCAAGTGAGAATCTCTTTGTCA-3’

R: 5’-CAGATCTCTGATGAATAACCAACG-3’; *Ae*. *aegypti* Actin: F: 5’-GAACACCCAGTCCTGCTGACA-3’, R: 5’-TGCGTCATCTTCTCACGGTTAG-3’

### Mosquito transfection

DNA plasmids were injected according to our published whole-body transfection method [84]. Briefly, Cellfectin II (Invitrogen, CA) was mixed with S2 Schneider’s medium at a 1:1 ratio and then keep at RT for 10 min. Plasmid DNA was combined with this mixture and incubated at RT for 30 minutes before thoracic microinjection into *Aedes aegypti*. Mosquitoes were injected with 500ng plasmid/300 nL solution. The dsRNA was injected as previously described and as indicated in the figure legends.

### Immunofluorescence analysis

Aag2 *Ae*. *aegypti* cells were infected with DENV at an MOI of 0.1. At 24 hours post-infection, infected cells and control cells were fixed in 4% paraformaldehyde for 20 min at RT, washed with PBS(-) and then stained for infection using antibodies against CRVP379 (L2 Diagnostics, CT), DENV envelope gene (Millipore, MA) and/or prohibitin (Abcam, MA). The antibodies were diluted in 1% BSA at 1/250 and cells were incubated for 20 minutes at RT. Any secondary antibodies used were standard (anti-mouse or anti-rabbit TRITC and FITC, DAPI and phalloidin), and were diluted according to manufacturer’s instructions. Infection was visualized using fluorescent microscopy, equipment and specifics can be found in figure legends.

### TAP expression plasmid constructs

All plasmids were prepared using Qiagen miniprep kits (Valencia, CA) after standard transformation into DH5α competent bacterial cells. The tagged virus protein nTAP expression plasmids were made by cloning the coding regions for each viral protein into the N-terminal TAP plasmid (Stratagene, CA).

### Western blots

Solutions were run on a 4–12% SDS-PAGE gel for 1.5 h at 15 milliamps per gel (unless figure legend indicates otherwise). The proteins were then transferred to PVDF membrane. The membrane was blocked with 5% milk in 1% TBST for 1h at RT and then incubated with the appropriate primary antibody overnight at 4°C. The membrane was washed and then incubated with the appropriate horseradish peroxidase secondary antibody for 1h at RT. The protein blots were incubated with ECL substrates (Amersham, NJ) for 5 min at RT and then detected on Kodak film. Antisera production is described below and prohibitin antibody was purchased from Santa Cruz.

### Transfection of plasmids (cells)

The expression plasmids were made from pAc5.1/V5-His A vector (Invitrogen, CA) and cloning was done using PCR along with gene-specific primers as previously described [85]. We used the Qiagen mini-prep kit to isolate DNA from bacterial cultures after transforming DH5-alpha cells. Plasmids were transfected into cells using Effectene (Qiagen, CA) according to manufacturer’s instructions. Briefly, for a 10 cm^2^ plate, 10 μg of DNA was mixed with 500 μL buffer EC and 32 μL enhancer was added. This was allowed to incubate for 5 min on the benchtop. Then, 30 μL Effectene reagent was added and the solution vortexed briefly. After 10 min incubation, the solution was added to the cells.

### TAP assay

The TAP assay was used to identify mosquito cell proteins that interacted with CRVP379 protein. All steps were done at 4°C to maintain the protein interactions. The cell or tissue lysates were applied to streptavidin resin, incubated at 4°C for 2 h, washed, and bound proteins eluted off. A second purification step was done with calmodulin resin and the proteins were boiled off into PBS(-). The eluted proteins were analyzed at the Yale University W.M. Keck Foundation core facility. The eluate was subjected to trypsin digestion followed by LC/MS-MS (liquid chromatography and mass spectometry) for peptide sequencing and identification using the recently completed *Aedes aegypti* mosquito genome [47]. Putative mosquito proteins were identified via amino acid sequence identity to both known mosquito proteins and their mammalian counterparts using the BLAST software on the NCBI website. Mosquito proteins found to bind the tags alone as well as proteins found to bind tagged green fluorescent protein were eliminated as putative interacting partners.

### Protein and antisera production

A recombinant protein consisting of residues 21–128 of CRVP379 was synthetically cloned into the pGEX-6p-1 expression vector (GE Life Sciences) into the BamH1 and Xho1 sites. The recombinant plasmid was transformed into Rosetta DE3 pLys2 *E*. *coli* cells. GST-CRVP379 protein was purifed from the bacteria cells as inclusion bodies by passing the E coli cells through a cell disruptor at 20 psi of pressure. Inclusion bodies were used to immunize rabbits to generate polycolonal antisera (CoCalico Biologicals, Reamstown, PA). Prior to immunization, rabbits were bled to obtain pre-immune control sera.

### ELISA analysis

Serum samples were coated onto a 96-well ELISA plate (Thermo Fisher Sci, MA) and incubated overnight at 4°C. The plate was blocked with 1% BSA in PBS(-) and incubated with recombinant CRVP for an hour at RT. The proteins were washed off, antibodies were added for 30 min at RT, washed off and secondary-HRP was added for 30 min at RT, washed off and TMB substrate was added for 20 min at RT. Stop solution was added and the O.D. of the wells was read at 450 nm.

## Supporting Information

S1 Fig List of mosquito genes and siRNA sequences used in RNAi experiments. (PDF)

S2 Fig Full results of siRNA screen on DENV infection in mosquito cells. (PDF)

S3 Fig Alignment of *Ae*.*aegypti* AAEL000374 & AAEL000379. (PDF)

S4 Fig Transfection of GFP control in Aag2 cells. (PDF)

S5 Fig Effects of Prohibitin overexpression on DENV infection in Aag2 cells with reduced CRVP379 expression. (PDF)

S6 Fig CRVP379 antibody staining in Aedes aegypti midguts with and without CRVP379 RNA intereference. (PDF)
