# Supplementary material for: Dengue Virus Infection of Aedes aegypti Requires a Putative Cysteine Rich Venom Protein
Source: PLoS Pathog. 2015 Oct 22;11(10):e1005202. doi: 10.1371/journal.ppat.1005202 (PMC4619585; doi:10.1371/journal.ppat.1005202)
Supplement: S1 Fig — (PDF) [file ppat.1005202.s001.pdf]

SeqID	GENE	Sense siRNA
AAEL006536-RA	hypothetical 1	GCU CAA GUG UCU ACA GAA ATT
		GCC UCA GAA UCG CUU CGU ATT
AAEL008415-RA	hypothetical 2	GGA CAA ACU CAA AGA GGA UTT
		GGA AGA GCU AAC UAG ACA UTT
AAEL009255-RA	hypothetical 3	GAA CAU CUA CGG UAU AAC ATT
		CAU CUA CGG UAU AAC AGG ATT
AAEL002477-RA	hypothetical 4	GCG CUC UAG GAA UAC UAU ATT
		GCU GCA UAA GUU GGG UCU ATT
AAEL014205-RA	conserved hypothetical 1	GCC GAA AGG AUU UAU CCA UTT
		GAG AAG ACC UUA CAC ACC ATT
AAEL012959-RA	conserved hypothetical 2	GGG CGA GCU UCU AAC UCA ATT
		CCA GCG AGA CAU GAU CAA ATT
AAEL004555-RA	hypothetical 5	GAU UAU UCU CUG ACU GAU ATT
		GAU UGA AAC AGA CGA UUA UTT
AAEL001156-RA	conserved hypothetical 3	GAU GUG GAC UAU ACG GAA ATT
		GCA AGA UAU UUC CCG ACA ATT
AAEL013288-RA	conserved hypothetical 4	GAA CAG ACA UUG AGA CGA CTT
		GCC UUG UUC UCA GCU GUU UTT
AAEL008835-RA	hypothetical 6	GGU GAA CUC UGU UUG CCA ATT
		GCU GAA AUG AAU UCC GAA ATT
AAEL009504-RA	hypothetical 7	CCA AGC GAU UUG CCA ACA ATT
		GGA AGU AUG CAU UGU UGU UTT
AAEL003685-RA	histone H3	GGA AGC CAG CGA AGC UUA UTT
		GAA GCC AGC GAA GCU UAU UTT
AAEL004593-RA	hypothetical 8	GCA ACA CCU ACU GUC GCA UTT
		GCA GAC GAG AAU ACG CCA ATT
AAEL014440-RA	juvenile hormone-inducible	GCG AAA UAC UGC CAG CAA UTT
		GGA GCA UGC UGU GCA ACA UTT
AAEL005312-RE	conserved hypothetical 5	GGA AUC AUC ACU AGU UGA ATT
		GCG CAG UUG AAA GCU UUA UTT
AAEL004555-RB	hypothetical 9	GCG AGG ACA ACU CUG AUU ATT
		GCU CUA UGG CAG UAU CUA ATT
AAEL010660-RA	alpha-B-crystallin, putative	CCA ACU UCG CAG AAG ACA ATT
		UCC GAG ACA CAG GAU CCA ATT
AAEL009263-RA	conserved hypothetical 6	GGA AUC AAA CUG CCU UGA UTT
		GCU GGA AUC AAA CUG CCU UTT
AAEL009491-RA	hypothetical 10	GCG AUG ACU GUG UCA UCA UTT
		GCU CUC UAA UUA UCG UAG UTT
AAEL008308-RA	conserved hypothetical 7	UCA UCG GAA CCC UUC CGA UTT
		CCA UCA GGU CUG UGU CCA UTT
AAEL007584-RA	conserved hypothetical 8	GCG CCA GUA CUG UCC AAU ATT
		GGG AAG CCU UCG ACU CAA UTT

AAEL013168-RA	arrowhead	CCG UAU AGU AGA AAC GAA UTT<br>GGU AAC AGG UUU GAG UAA ATT
AAEL005153-RA	hypothetical 11	CCC AUC CUA AUG UUU ACU UTT<br>GGC CAA UCG UUU GGU AGA ATT
AAEL004104-RA	hypothetical 12	GCA ACC GGC GAA CAA AUU UTT<br>GCU GCA GGA CUU ACU GAA ATT
AAEL014276-RA	conserved hypothetical 9	GCG UCG AUU UGG CUC AAU UTT<br>GGC CAU CGG CAG AAC UAU UTT
AAEL006354-RA	epoxide hydrolase	GCG AUC CAG GCC GUU AUU ATT<br>CCA UUA UGC CGA GGU UAA UTT
AAEL001958-RA	hypothetical 13	GCA GGA GAC AUC ACU CCA ATT<br>CCA GAA GUC CCG UUU ACA ATT
AAEL014446-RA	hypothetical 14	AUU UAA AUC CGU UGG ACA ATT<br>GUG CAU AAA CUG AAU CCA UTT
AAEL007025-RA	conserved hypothetical 10	GCU CCU CAU GGA AUC GAA ATT<br>GCA GUU AUU CCU CAA GUA UTT
AAEL005938-RA	hypothetical 15	GCC UGA CUG UAA AGU CGA UTT<br>GCA ACA UUA CAA UGC UCA UTT
AAEL011141-RA	hypothetical 16	GGA CGA CGG AGG UAA CAA UTT<br>GCU UCA UAU GUG GCA AGC UTT
AAEL005923-RA	conserved hypothetical 11	GGA UUG UGC GAU GUU CGA UTT<br>GCC AGU CUA GGU CCA GUA ATT
AAEL004115-RA	hypothetical 17	GCC AAC UCC CAG GAC AAU UTT<br>CCC AGG ACA AUU GCA AUU UTT
AAEL006827-RA	cytochrome P450 1	GCU UCU UCC GCG ACA ACU ATT<br>GCG GUU GAU UAC GGU GCU UTT
AAEL012551-RA	conserved hypothetical 12	GGA GGU GUA UUA UGC GGA ATT<br>GGA GGA UCA AAG CCA UGA ATT
AAEL009762-RA	cytochrome P450 2	CCG GAA CGG UAG UCU UCA UTT<br>GCC GAG ACA CCA UUA UGU ATT
AAEL008903-RA	zinc finger protein	GCC GUU GCU AAU GGA GUA UTT<br>CCG CAC AGU UGU GAC CUU UTT
AAEL002330-RA	conserved hypothetical 13	CCC GAU CCC AAU UCG AGA ATT<br>CCG GUG AAG UAC UCU AUA ATT
AAEL002743-RA	synaptic vesical protein	GCA AUG GCU UAC AUA GCA UTT<br>CCC AGA ACU CAU GAA UCA ATT
AAEL004139-RA	hypothetical 18	GCA GCC ACU AAU GGA GCU ATT<br>GCC ACU AAU GGA GCU AGU UTT
AAEL009768-RA	hypothetical 19	UCU GUC GGU GGU GUG CUA UTT<br>GAU GUG UAC AGC AUU CGA ATT
AAEL011669-RA	hypothetical 20	GAG CCU AAA UCA UCC GGU UTT<br>GCC GUU AAG AUC AUU UCC ATT
AAEL007855-RA	hypothetical 21	GCA CGA AGU GAA GGU ACG UTT

		GGA AAG CUU ACU GGA GAU UTT
AAEL007588-RA	hypothetical 22	GCU CAA UGC UCU GCC GAU UTT
		CCG CUU UAA ACG UUC AAA UTT
AAEL000379-RA	cysteine-rich venom, putative	GCG GUA AUU GCG CUA AUC UTT
		GCU GGU AAU ACC CGU ACC ATT
AAEL006146-RA	hypothetical 23	CUG UCC UAC CUG AGG CAA ATT
		CCU GAG AUU CGC AAG CGU ATT
AAEL013288-RB	conserved hypothetical 14	CCU ACC UAU CUU AGC CAU UTT
		GCC UUG UUC UCA GCU GUU UTT
AAEL006123-RA	trypsin, putative	CCG UUA AGU CAU GCC AAA UTT
		CCA AUU CGG UUA GGC CCA UTT
AAEL009962-RA	hypothetical 24	GCA ACG UAU UGU CUU GCA ATT
		GCA AAU ACC GUA AGG AGA ATT
AAEL010910-RA	retina abberant	CCG CUU GUU AAA UUG CCU UTT
		GGU UGU UUC CUA CGC CUA UTT
AAEL005358-RB	conserved hypothetical 15	GGA AUA AUG CAU AUC CUA UTT
		CGG AAU AAU GCA UAU CCU ATT
AAEL009973-RA	hypothetical 25	GCU GAU AAU AGG AAA UAU GTT
		GAG CCG UGG ACG UUG UCU ATT
AAEL004861-RA	peroxisomal integral membrane Per 8p	GGA AGC GGA CAU UAU UCA ATT
		GGG CAU UUC CUG UCC CAU ATT
AAEL003339-RA	hypothetical 26	CCU CAU UAU CCA CCU CAA UTT
		CCC UCA UUA UCC ACC UCA ATT
AAEL010291-RA	hypothetical 27	GCU CUU CCG CUC AUG CAA UTT
		GCC UAU GAU CCA GUA UGA ATT
AAEL012834-RA	hypothetical 28	AGA AAG UCC ACA AGG UUC ATT
		GGA GAA AGU CCA CAA GGU UTT
AAEL009683-RA	hypothetical 29	GCU CGC AAC GCU UCU AAU ATT
		GCG GUC UUC AUC GCU AAU UTT
AAEL015163-RA	cuticle, putative 1	CCA GAG UAC UCC UAC AGC UTT
		CAU CCA GAG UAC UCC UAC ATT
AAEL014338-RA	hypothetical 30	GGA AAU GUA UUA CAC CAA CTT
		CCC AGG ACG UUU CUU UGG ATT
AAEL000183-RA	hypothetical 31	CCA GUA AUA GCC GAA UCC UTT
		GCC GAA UCC UAU UCA CAC UTT
AAEL012834-RA	hypothetical 32	GGA GAA AGU CCA CAA GGU UTT
		AGA AAG UCC ACA AGG UUC ATT
AAEL009800-RA	cuticle, putative 2	UGA CGA AUA CGA CCC ACA UTT
		CAG AGU ACU CCU ACA GCU ATT
AAEL007591-RA	conserved hypothetical 16	GUU GGA AGU GCC AGC GCC ATT
		UGA CGA AUA CGA CCC ACA UTT
AAEL004157-RA	hypothetical 33	GCC GAG UUC CAA CAA AUA ATT
		GCU UGG UAA UCG CCG UUG UTT

AAEL002241-RA	cuticle, putative 3	GCU UAC GCU UAU CAA CCA ATT
		UCG CCG UUG UUG CCC AGA ATT
AAEL013284-RA	serine-type enodpeptidase	CCC ACU GUU CGC UAC GAA UTT
		UCG CAU CUC UGA UGG CUU UTT
AAEL000443-RA	conserved hypothetical 17	ACA CGG AGC GCA UUA AAU ATT
		GGU GGU CAG UGC UAU GCA UTT
AAEL004761-RA	serine/threonine-protein kinase MAK	GGG AAA UAC CAA AGU GGA ATT
		GCU CUU GAA CCG AGG UCU UTT
AAEL010291-RA	hypothetical 34	GCU CUU CCG CUC AUG CAA UTT
		GCC UAU GAU CCA GUA UGA ATT
AAEL009989-RA	hypothetical 35	GGA AUU UGU GCG CUA UUA UTT
		GCU AUG UGG AGU GAC UAU UTT
AAEL013944-RA	hypothetical 36	GGA UGA GUG UUU UGA GUG ATT
		AAA CAA UCA GAA AGC CCA ATT
AAEL013738-RA	hypothetical 37	UCA GAC GGG CAU GAU CGA ATT
		GAU GCG UUC GAU UGA GAU UTT
AAEL005838-RA	hypothetical 38	GCG CCC AAG UUU GAA GCU UTT
		GCG AAA UUC CCG UGC GAA UTT
AAEL004170-RB	hypothetical 39	CCU ACG UAA GGC UUG UUA ATT
		GGG AUU UGC AUU GAA GCA ATT
AAEL001408-RA	hypothetical 40	GCC AUU AAC AUC AAA CCC UTT
		GGA CUG AAG CUA ACU GUA UTT
AAEL014793-RA	hypothetical 41	GGG CAA UAU AUG AAA CCA ATT
		GAC UAG GAC UGG UGU ACU UTT
AAEL002326-RA	pickpocket, putative	ACA CGG AGC ACA UUA AAU ATT
		CCA GAG CUC UUG AGU ACA UTT
AAEL002224-RA	hypothetical 42	GGA CAA CUU AUA CGU GCA ATT
		GGA AGG GAC UUA UGG ACA ATT
AAEL010436-RA	hypothetical 43	UCU UCU AUU CUU CGA CGA ATT
		GUU CAA CUC UUU UCC GAU UTT
AAEL007013-RA	RNA-binding precursor, putative	GAC AAU AGG CUG CGG GAA UTT
		AGA GAG AAG AAA GGA GUA ATT
AAEL004170-RA	hypothetical 44	AAU CAG AGG UGG AUA CAA UTT
		CAA AUG AAG UGA AAC UAG ATT
AAEL013577-RC	hypothetical 45	UGC AAU AUC UCG UCA GCG ATT
		CUU GAG AAC UGG AGG AUU GTT
AAEL007324-RA	hypothetical 46	GAA ACA ACC CGA CCA GUC ATT
		CGA CCA GUC AAU UGC AAC ATT
AAEL014084-RA	KIF11	GGA CGC CAC UAA CUU GGA UTT
		GGA ACU GAC GCU GUA UCA ATT
AAEL014900-RA	ATM	GCA AAC ACU GUU GCC AAU ATT
		GCU CAA UCC CUG UAC CAU ATT
AAEL008123-RA	PRKDC(DNK-PK)	CCA AAU AUC CGU CUG ACA ATT

		GCU GAA CAC GCA CUC GUU ATT
AAEL010069-RA	ATR	GGA GUU UAC CAU UCU UCA ATT
		GCU CCA AUA GUG AUC CAA ATT
AAEL005809-RA	CEP290	AGA AUG ACC UAA AGA GAA ATT
		CAA UGG AGU UAG AGC GCA ATT
AAEL006121-RA	F2: coagulation factor II (thrombin)	GCU UUC CAU CGC UCU AAA UTT
		CCA UCG CUC UAA AUG AUA ATT
AAEL009851-RA	REV3L (DNA polymerase zeta)	GCU UAU CUC AAU CGA GUA ATT
		GCG CUU GAA GCA UGU UCU ATT
AAEL005888-RA	POLQ DNA polymerase theta	GCA GGU UGC GUC CAC AUU UTT
		GCU GCG GAA UUA CAC CAA ATT
AAEL011098-RA	NMEK7 (NDPK 7)	GCA CGU UGU GUA UCA UCA ATT
		GGA UUC AUA AUU UCG GCA ATT
AAEL015671-RA	POLG DNA polymerase gamma	CCG AGA GCG CUA UGU UCA ATT
		GGA ACG CAU UGG UAG UGA ATT
AAEL011825-RB	aryl hydrocarbon receptor	AUU UAA AUC UCG AAC GGA ATT
		AUU UAA AUC UCG AAC GGA ATT
AAEL014251-RA	BIRC5 (survivin)	GGA AAU GAC GUG UGA GGA ATT
		GAA AUG ACG UGU GAG GAA ATT
AAEL001983-RA	ZNF217: zinc finger protein 217	GCC CAA AGU GCG GUA AGA UTT
		GGU GAC UGG CAU GAA CCA ATT
AAEL003201-RA	CEL: carboxyl ester lipase	GCA AUU UGC AGA ACG UUA UTT
		GGG CAU CCC AUA UGG UGA ATT
AAEL007544-RA	Chk2	GGU CAA AGA CAC GUA CUA UTT
		GCU GAU GAA UGU GCG GCU UTT
AAEL001684-RA	BRCA2	GGG ACC GUA AGC UAA ACA UTT
		GCA AGU AAG UUG GCA CCA UTT
AAEL013353-RA	profilin partial mRNA	AGG CUG TTA TAG TAT CAA UTT
		CGU CUA UAG UCG AAA AGC UTT
