# Supplementary material for: Dengue Virus Infection of Aedes aegypti Requires a Putative Cysteine Rich Venom Protein
Source: PLoS Pathog. 2015 Oct 22;11(10):e1005202. doi: 10.1371/journal.ppat.1005202 (PMC4619585; doi:10.1371/journal.ppat.1005202)
Supplement: S2 Fig — (PDF) [file ppat.1005202.s002.pdf]

Gene Name	SeqID	% Control Infection
hypothetical 1	AAEL006536-RA	34.78%
hypothetical 2	AAEL008415-RA	34.40%
hypothetical 3	AAEL009255-RA	34.15%
hypothetical 4	AAEL002477-RA	41.16%
hypothetical 5	AAEL004555-RA	27.18%
hypothetical 6	AAEL008835-RA	85.21%
hypothetical 7	AAEL009504-RA	69.29%
hypothetical 8	AAEL004593-RA	61.52%
hypothetical 9	AAEL004555-RB	127.35%
hypothetical 10	AAEL009491-RA	43.58%
hypothetical 11	AAEL005153-RA	129.92%
hypothetical 12	AAEL004104-RA	129.59%
hypothetical 13	AAEL001958-RA	101.61%
hypothetical 14	AAEL014446-RA	136.78%
hypothetical 15	AAEL005938-RA	56.48%
hypothetical 16	AAEL011141-RA	34.13%
hypothetical 17	AAEL004115-RA	109.82%
hypothetical 18	AAEL004139-RA	81.16%
hypothetical 19	AAEL009768-RA	59.61%
hypothetical 20	AAEL011669-RA	40.14%
hypothetical 21	AAEL007855-RA	47.63%
hypothetical 22	AAEL007588-RA	44.00%
hypothetical 23	AAEL006146-RA	127.85%
hypothetical 24	AAEL009962-RA	33.08%
hypothetical 25	AAEL009973-RA	76.90%
hypothetical 26	AAEL003339-RA	25.76%
hypothetical 27	AAEL010291-RA	17.82%
hypothetical 28	AAEL012834-RA	16.19%
hypothetical 29	AAEL009683-RA	15.59%
hypothetical 30	AAEL014338-RA	15.84%
hypothetical 31	AAEL000183-RA	19.59%
hypothetical 32	AAEL012834-RA	24.77%
hypothetical 33	AAEL004157-RA	20.39%
hypothetical 34	AAEL010291-RA	14.85%
hypothetical 35	AAEL009989-RA	10.77%
hypothetical 36	AAEL013944-RA	19.11%
hypothetical 37	AAEL013738-RA	23.95%
hypothetical 38	AAEL005838-RA	23.41%
hypothetical 39	AAEL004170-RB	17.37%
hypothetical 40	AAEL001408-RA	21.52%
hypothetical 41	AAEL014793-RA	20.24%
hypothetical 42	AAEL002224-RA	18.52%
hypothetical 43	AAEL010436-RA	16.14%
hypothetical 44	AAEL004170-RA	17.77%
hypothetical 45	AAEL013577-RC	23.13%
hypothetical 46	AAEL007324-RA	25.76%
conserved hypothetical 1	AAEL014205-RA	23.02%
conserved hypothetical 2	AAEL012959-RA	70.17%
conserved hypothetical 3	AAEL001156-RA	128.57%
conserved hypothetical 4	AAEL013288-RA	90.61%

conserved hypothetical 5	AAEL005312-RE	93.14%
conserved hypothetical 6	AAEL009263-RA	31.84%
conserved hypothetical 7	AAEL008308-RA	93.22%
conserved hypothetical 8	AAEL007584-RA	125.98%
conserved hypothetical 9	AAEL014276-RA	110.78%
conserved hypothetical 10	AAEL007025-RA	154.78%
conserved hypothetical 11	AAEL005923-RA	82.48%
conserved hypothetical 12	AAEL012551-RA	158.59%
conserved hypothetical 13	AAEL002330-RA	106.14%
conserved hypothetical 14	AAEL013288-RB	160.59%
conserved hypothetical 15	AAEL005358-RB	26.59%
conserved hypothetical 16	AAEL007591-RA	23.11%
conserved hypothetical 17	AAEL000443-RA	21.43%
alpha-B-crystallin, putative	AAEL010660-RA	55.77%
cytochrome P450 2	AAEL009762-RA	26.38%
zinc finger protein	AAEL008903-RA	56.32%
cysteine-rich venom protein	AAEL000379-RA	44.61%
histone H3	AAEL003685-RA	44.14%
juvenile hormone-inducible	AAEL014440-RA	136.78%
arrowhead	AAEL013168-RA	153.98%
epoxide hydrolase	AAEL006354-RA	125.46%
cytochrome P450 1	AAEL006827-RA	123.73%
synaptic vesical protein	AAEL002743-RA	101.97%
retina abberant	AAEL010910-RA	38.89%
trypsin, putative	AAEL006123-RA	22.54%
peroxisomal integral membrane Per 8p	AAEL004861-RA	20.78%
cuticle, putative 1	AAEL015163-RA	15.50%
pickpocket, putative	AAEL002326-RA	22.56%
RNA-binding precursor, putative	AAEL007013-RA	32.10%
KIF11	AAEL014084-RA	30.29%
ATM	AAEL014900-RA	22.22%
PRKDC(DNK-PK)	AAEL008123-RA	16.30%
cuticle, putative 2	AAEL009800-RA	21.31%
CEP290	AAEL005809-RA	25.41%
ATR	AAEL010069-RA	19.50%
cuticle, putative 3	AAEL002241-RA	27.66%
serine-type enodpeptidase	AAEL013284-RA	28.29%
REV3L (DNA polymerase zeta)	AAEL009851-RA	33.08%
POLQ DNA polymerase theta	AAEL005888-RA	185.28%
NMEK7 (NDPK 7)	AAEL011098-RA	307.74%
serine/threonine-protein kinase MAK	AAEL004761-RA	17.07%
F2: coagulation factor II (thrombin)	AAEL006121-RA	Not determined
POLG DNA polymerase gamma	AAEL015671-RA	Not determined
aryl hydrocarbon receptor	AAEL011825-RB	Not determined
BIRC5 (survivin)	AAEL014251-RA	Not determined
ZNF217: zinc finger protein 217	AAEL001983-RA	Not determined
CEL: carboxyl ester lipase	AAEL003201-RA	Not determined
Chk2	AAEL007544-RA	Not determined
BRCA2	AAEL001684-RA	Not determined
profilin partial mRNA	AAEL013353-RA	Not determined
