# Supplementary material for: Dengue Virus Infection of Aedes aegypti Requires a Putative Cysteine Rich Venom Protein
Source: PLoS Pathog. 2015 Oct 22;11(10):e1005202. doi: 10.1371/journal.ppat.1005202 (PMC4619585; doi:10.1371/journal.ppat.1005202)
Supplement: S3 Fig — (PDF) [file ppat.1005202.s003.pdf]

```
AAEL000374      MRFIIGVVFCLAL---ALAIVGAQRANQEYKECGSACPPTCESIKREPMMCIAQCKSGWF
AAEL000379      MKLLISLAVIALIYTCVTASNFCSGPNEVYQECGSACEKTCAGLGAN-QTCNEKCVPGCF
                *:::*.:..   :   . *   ..  *: *:******  ** .:  :   *  :*  * *

AAEL000374      CKSGYVR-NAAGKCVKPSQCPK--------------------------------------
AAEL000379      CADGFVRLNHSGQCVPSSKCPKVRVRRAPEPLPPLVIPVPIPIPIPVPPIVRPRGPLWWL
                * .*:** * :*:**  *:***

AAEL000374      ---------
AAEL000379      RPPPPPLFG
```
