# Supplementary figures and images for: Dengue Virus Infection of Aedes aegypti Requires a Putative Cysteine Rich Venom Protein
Source: PLoS Pathog. 2015 Oct 22;11(10):e1005202. doi: 10.1371/journal.ppat.1005202 (PMC4619585; doi:10.1371/journal.ppat.1005202)

Image of GFP-transfected Aag2 cells.

PHASE

GFP/FITC

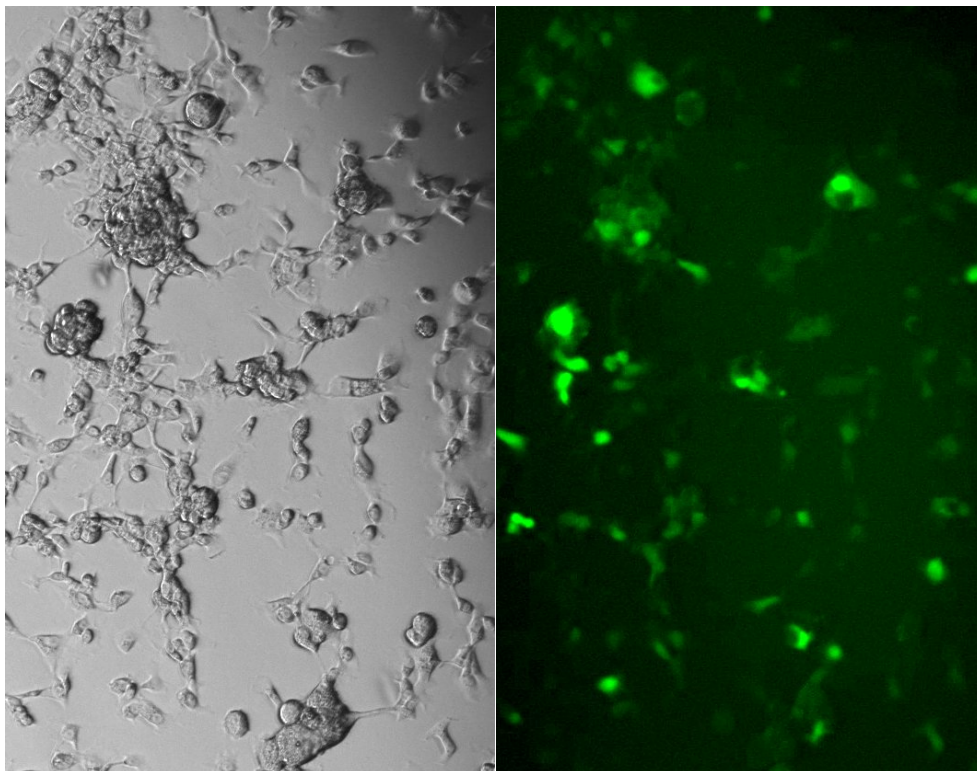

Supplement: S4 Fig — (PDF) [file ppat.1005202.s004.pdf]

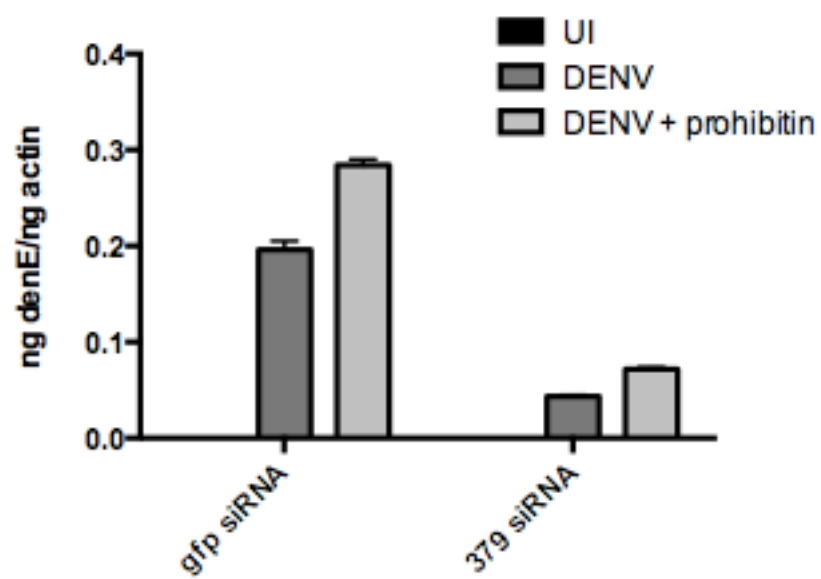

Supplement: S5 Fig — (PDF) [file ppat.1005202.s005.pdf]
