# Supplementary material for: Dengue Virus Infection of Aedes aegypti Requires a Putative Cysteine Rich Venom Protein
Source: PLoS Pathog. 2015 Oct 22;11(10):e1005202. doi: 10.1371/journal.ppat.1005202 (PMC4619585; doi:10.1371/journal.ppat.1005202)

Midgut of *Aedes aegypti* injected with dsRNA against coding region of CRVP379 protein. Stained with antisera against CRVP379.

PHASE Anti-CRVP379

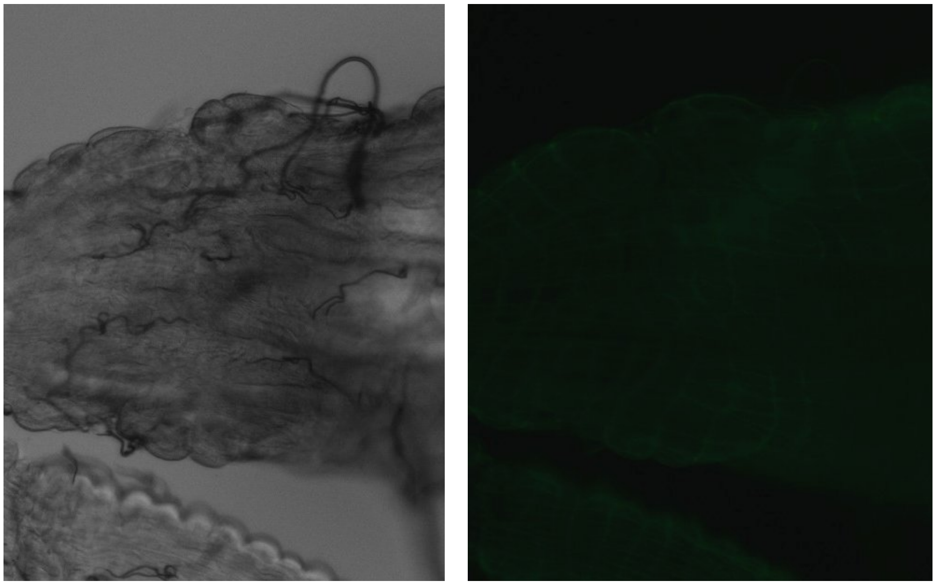

Supplement: S6 Fig — (PDF) [file ppat.1005202.s006.pdf]
